# Composite resection of the left upper lobe and superior segment (S6) of the lower lobe for lung cancer with a mediastinal lingular and basal pulmonary artery

**DOI:** 10.1186/s40792-018-0445-0

**Published:** 2018-04-20

**Authors:** Tomoyuki Nakano, Shigemi Ishikawa, Yasunori Sohara, Shunsuke Endo

**Affiliations:** 1 Department of Chest Surgery, International University of Health Welfare Hospital, Nasushiobara, Tochigi Japan; 2 Department of General Thoracic Surgery, Jichi Medical University, 3311-1 Yakushiji, Shimotsuke, Tochigi 329-0498 Japan

**Keywords:** Anatomical variation of pulmonary artery, Mediastinal pulmonary artery, Three-dimensional computed tomography

## Abstract

**Background:**

Preoperative evaluation and awareness of anatomical variations in the pulmonary vessel is essential for a secure pulmonary resection. We herein present a patient who underwent complex pulmonary resection for lung cancer with a mediastinal lingular and basal pulmonary artery that had been detected by preoperative three-dimensional computed tomography.

**Case presentation:**

The patient was an asymptomatic 66-year-old woman who had a 39-pack-year smoking habit. Chest computed tomography (CT) revealed the tumor invading the left upper bronchus and pulmonary artery branches in the left upper lung lobe. Enhanced CT and three-dimensional (3D) images of the pulmonary artery revealed that pulmonary artery branches (A4 + 5, A8, and A9 + 10) were extending into the lingular and basal segment in ventral side of the left upper bronchus. We completed the resection by means of a composite resection of the left upper lobe and the superior segment of the lower lobe, avoiding pulmonary angioplasty to preserve the left lower lobe or pneumonectomy.

**Conclusions:**

3D-CT is useful for detecting this rare variation of the left pulmonary artery before operation, allowing for proper resection.

**Electronic supplementary material:**

The online version of this article (10.1186/s40792-018-0445-0) contains supplementary material, which is available to authorized users.

## Background

There is some variation of the branching pattern of the pulmonary artery, and it is not uncommon for general thoracic surgeons to see this variation, particularly in the left lung. Variations appear more frequently in the left pulmonary artery, with the most common variations being the mediastinal lingular pulmonary artery [1]. We present a patient with lung cancer and an extremely rare mediastinal lingular and basal pulmonary artery. In this type of case, there is a risk of dividing the mediastinal basal pulmonary artery during the left upper lobectomy. In the case being reported, the operation was performed safely because we became aware of the variation after three-dimensional computed tomography (3D-CT) preoperatively.

## Case presentation

A pulmonary tumor was found on a routine chest X-ray of an asymptomatic 66-year-old woman. She had a history of cervical cancer and a 39-pack-year smoking habit. Laboratory examination revealed high serum levels of squamous cell carcinoma antigen (8.0 ng/ml; reference range, 0.0–1.5 ng/ml).

**Fig. 1:**
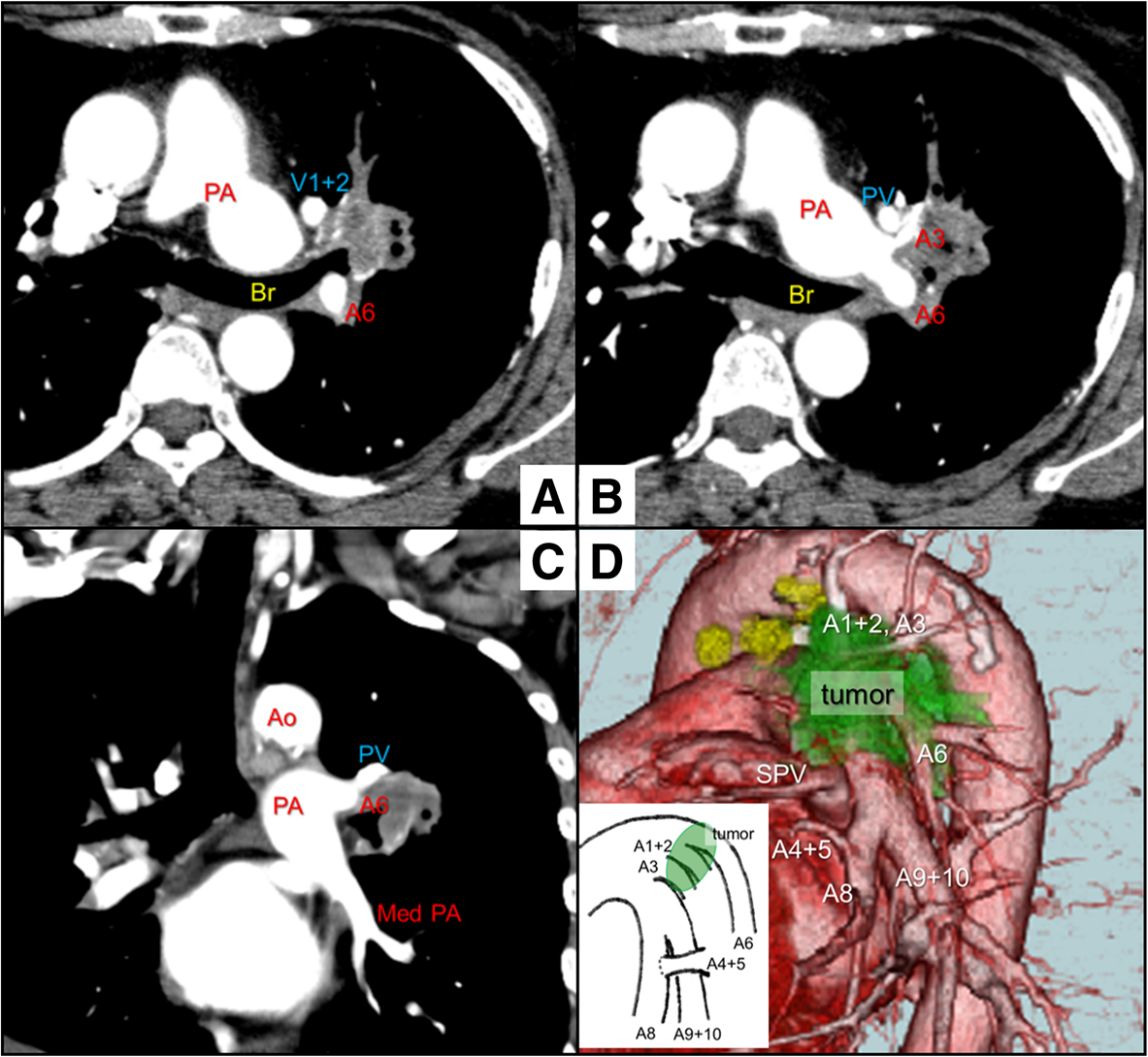
Chest computed tomography (CT) (**a**, **b** axial view and **c** coronal view) demonstrated the tumor invading the left upper bronchus in the left upper lung lobe, and the A3 and A6 branches were involved in the tumor. **d** Three-dimensional CT revealed that pulmonary artery branches (A4 + 5, A8, and A9 + 10) extended into the dorsal side of the superior pulmonary vein


CT demonstrated the tumor (3.0 × 2.0 × 2.0 cm) invading the left upper bronchus in the left upper lung lobe (Fig. 1a) and suggested that pulmonary artery branches were involved in the tumor with enlarged hilar lymph nodes (Fig. 1b, c). ^18^F-Fluorodeoxyglucose positron emission tomography (PET) revealed a maximum standardized uptake value of 6.1. Neither mediastinal lymphadenopathy nor distant metastasis was found on PET-CT. Bronchoscopic examination revealed that the superior segment bronchus of the left lung was obstructed by the tumor. In contrast, the left secondary carina and lower bronchus showed no abnormalities such as redness or irregular surface. No anomalous bronchial branches were found. Pathologic examination using a transbronchial biopsy specimen revealed the tumor to be a squamous cell carcinoma. We decided to perform the left upper lobectomy, and then, enhanced chest CT and 3D images of the pulmonary artery were constructed preoperatively. The horizontal sectional view of enhanced CT revealed that pulmonary artery branches (A4 + 5, A8, and A9 + 10) were extending into the lingular and basal segment in the ventral side of the left upper bronchus (Additional file 1: Video 1). Moreover, 3D-CT more clearly revealed the same pulmonary artery branches (Fig. 1d).

**Additional file 1:** **Video 1.** Horizontal sectional view of enhanced CT revealed that mediastinal A4 + 5, A8, and A9 + 10 branches were extending into the lingular and basal segment in the ventral side of the left upper bronchus. (WMV 4310 kb)


**Fig. 2:**
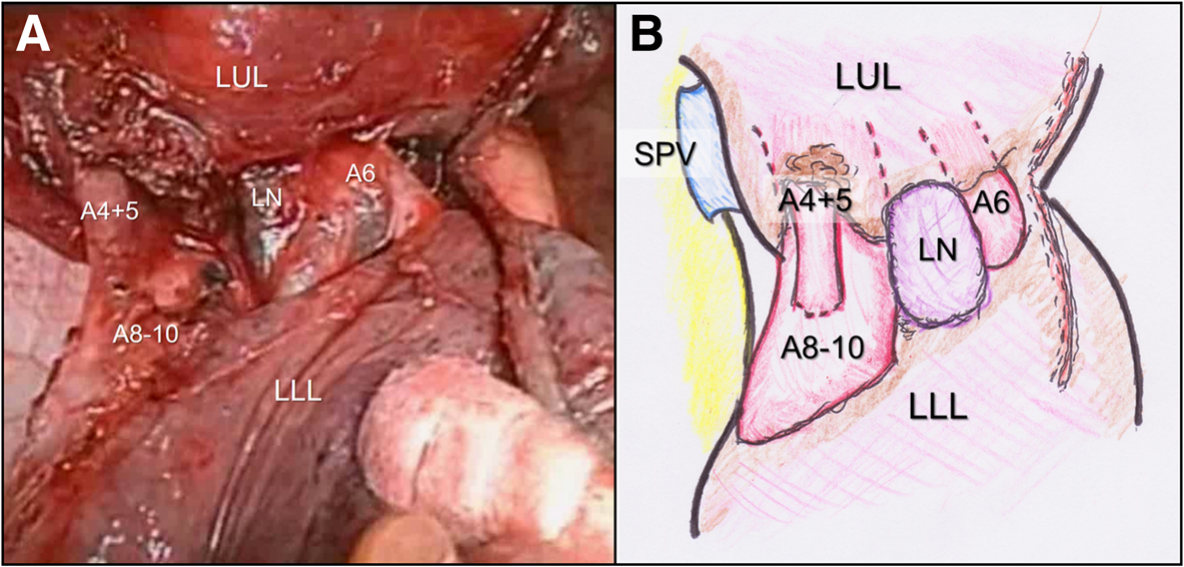
**a** Intraoperative findings (top, cranial side; left, ventral side) and **b** a schematic diagram. Mediastinal A4 + 5, A8, and A9 + 10 branches entered the lingular and basal segment. The A6 branch extended through the dorsal side separately


Induction chemotherapy was not considered because of the comorbid interstitial change in both lungs. The left upper lobectomy and mediastinal lymph node dissection were planned via posterolateral thoracotomy. During hilar dissection, enlarged lymph nodes involving the pulmonary artery were identified (Additional file 2: Video 2). We divided the inter-lobar fissure and found that one was the A6 branch at the dorsal side of the left main bronchus and the other was the mediastinal artery at the ventral side of the left main bronchus (Fig. 2). The branches of this mediastinal artery entered the lingular and basal segment. We could not preserve the A6 branch because its orifice was involved in the tumor. Therefore, we transected the A1-3 and A6 branches in a bundle and performed S6 segmentectomy in addition to the left upper lobectomy. The hilar bronchus was not involved in the tumor and lymph nodes so that the left upper bronchus and B6 branch could be easily transected with staplers. As a result, we could perform complete cancer resection, avoiding pulmonary angioplasty to preserve the left lower lobe or pneumonectomy. Preoperative CT imaging enabled a safe operation without damaging exceptional pulmonary artery branches.

**Additional file 2:** **Video 2.** Intraoperative findings (top, cranial side; left, ventral side). Mediastinal A4 + 5, A8, and A9 + 10 branches were extending into the dorsal side of the superior pulmonary vein. The A1-3 and A6 branches were transected in a bundle and S6 segmentectomy in addition to the left upper lobectomy was performed. (WMV 32529 kb)


The tumor was pathologically diagnosed as squamous cell carcinoma with subaortic lymph node involvement. Resection stumps of bronchus and vessels were not histopathologically involved in tumor cells, and the surgical margin was negative for cancer. The pathological stage was confirmed to be T1bN2M0 in accordance with the seventh edition of the TNM classification from the Union for International Cancer Control. The patient’s postoperative course was uneventful. She underwent adjuvant chemotherapy and, at the time of this writing, has been alive without recurrence for more than 5 years after the operation.

### Discussion

Pulmonary vascular variations are not uncommon in lung surgery. Sobotich et al. [1] reported that 16.4% of patients have pulmonary vessel variations, and most of the variations of the pulmonary artery appear in the left lingular artery. The most common variation is the mediastinal lingular artery (including the A4 + 5 type and either the A4 or A5 type), which has been reported in 27.3% of cases [2]. However, the mediastinal basal artery is an extremely rare variation. Even rarer, as in our case, is when the mediastinal artery is composed not of the partial lingular and basal artery, but of the total lingular (A4 + 5) and basal artery (A8, A9, A10) [3–5]. Rare left pulmonary artery variations place patients at risk of fatal impact by provoking misguided vessel transection, especially in thoracoscopic surgery. Through accurate preoperative awareness of pulmonary artery branches, the mediastinal lingular artery can be ligated properly without dividing the mediastinal basal pulmonary artery during the left upper lobectomy. A previous report suggested that 3D-CT is useful for detecting variation in pulmonary vessels and should be performed before lung surgery [4–6]. Virtual lung anatomical information provided by 3D images enables detailed surgical simulation and causes decrease of vascular injuries and efficient performance. Hagiwara et al. reported the accuracy and usefulness of high-quality 3D-CT angiography [6]. In that report, 3D-CT was performed in 124 patients who underwent thoracoscopic anatomical lung resection, pulmonary artery branches were precisely identified for 97.8% (309 of 316) of branches on 3D images, and patients undergoing preoperative 3D-CT tended to have lower incidences of postoperative complications and have significantly shorter operative time than those without 3D images. The undetected branches were limited to bilateral upper lobe, and the sizes of the branches ranged from 1 to 2 mm. The limitation of 3D-CT is identifying small vessels with an anatomically complex overlap of pulmonary artery and vein branches. Further advances in 3D-CT imaging technology including 3D software will be useful in the further development of surgery. Alongside other issues, 3D-CT is expensive in cases of re-examination for more detailed image data and is a time-consuming procedure.

## Conclusions

3D-CT should be considered when recommending for use on all patients in terms of cost and time. We thus suggest that 3D-CT should be performed only for patients who expect to undergo lobectomy or segmentectomy of the left upper lobe and are suspected of having a mediastinal pulmonary artery on a horizontal sectional view of CT.

## Abbreviations

LN: Lymph node
PET: Positron emission tomography
